# The Compensation of Expert Witnesses

**Published:** 1881-12

**Authors:** 


### THE COMPENSATION OF EXPERT WITNESSES.

A collection of decisions was not long since made by Dr. Stanford E. Chaille on the question, whether a court can force a medical expert to testify without securing him adequate compensation.

The following facts are pertinent to this question: English courts have decided that a scientific expert need not attend a subpœna, that his testimony cannot be forced, and that he must be compensated. In 1877 the Supreme Court of Alabama decided to the contrary; but, also in 1877, a Circuit Court of West Virginia concurred in the English view; in 1878, Judge

Clark, in the case of the "State of Texas *vs.* Jasper Weathers," decided that he "knew of no law to force a physician to attend court, and testify as an expert, without compensation;" and also in 1878, the Supreme Court of Indiana, reversing the decision of a lower court, maintained the expert's right to compensation. The Iowa code of 1873, page 593, sec. 3814 (and probably the laws of some other States), wisely provides that "witnesses called to testify only to an opinion, founded on special study or experience in any branch of science, or to make scientific or professional examinations, and to state the resul s thereof, shall receive additional compensation, to be fixed by the court, with reference to the value of the time employed, and the degree of learning or skill required."

This law grants all the medical profession demands, and its enactments by the General Assemblies of all the other States should be urged upon them by medical bodies.

---

Habitual Mouth Breathing, its Causes, Effects, and Treatment. By Clinton Wagner, M. D., Prof. Diseases of the Throat, Univ. of Vermont, &c., &c. New York, G. P. Putnam's Sons, 1881. San Francisco, Billings, Harbourne & Co.

The arguments against mouth-breathing are well put in this little volume of 50 pages. Though we do not believe more than one half what is said against the habit, yet we are quite willing to recommend the reading of the work.

---

The Birth Rate in France is 26 per 1000 of population annually; in Germany 40 per 1000.

